# Geometry- and Length Scale-Dependent Deformation and Recovery on Micro- and Nanopatterned Shape Memory Polymer Surfaces

**DOI:** 10.1038/srep23686

**Published:** 2016-03-30

**Authors:** Wei Li Lee, Hong Yee Low

**Affiliations:** 1 Engineering Product Development, Singapore University of Technology and Design, 8 Somapah Road, Singapore 487372, Singapore; 2 Department of Materials Science and Engineering, Massachusetts Institute of Technology, 77 Massachusetts Avenue, Cambridge, MA 02139, United States

## Abstract

Micro- and nanoscale surface textures, when optimally designed, present a unique approach to improve surface functionalities. Coupling surface texture with shape memory polymers may generate reversibly tuneable surface properties. A shape memory polyetherurethane is used to prepare various surface textures including 2 μm- and 200 nm-gratings, 250 nm-pillars and 200 nm-holes. The mechanical deformation via stretching and recovery of the surface texture are investigated as a function of length scales and shapes. Results show the 200 nm-grating exhibiting more deformation than 2 μm-grating. Grating imparts anisotropic and surface area-to-volume effects, causing different degree of deformation between gratings and pillars under the same applied macroscopic strain. Full distribution of stress within the film causes the holes to deform more substantially than the pillars. In the recovery study, unlike a nearly complete recovery for the gratings after 10 transformation cycles, the high contribution of surface energy impedes the recovery of holes and pillars. The surface textures are shown to perform a switchable wetting function. This study provides insights into how geometric features of shape memory surface patterns can be designed to modulate the shape programming and recovery, and how the control of reversibly deformable surface textures can be applied to transfer microdroplets.

Stimuli-responsive materials, in particular, shape memory polymers (SMPs), are smart materials that are capable of memorizing temporary shapes and recovering their permanent shapes via external stimuli such as heat^1^,^2^, light^3^ and magnetic field^4^. Stimulus-responsive and shape memory properties are beneficial for applications in biomedical devices (e.g. surgical stents and sutures)^3^,^5^, temperature sensors^6^, self-healing materials^7^,^8^, smart adhesives^9^ and deployable structures^10^. Most SMPs consist of molecular netpoints (e.g. crosslinking or crystallization) or the hard segments, and switchable soft segments. In thermoplastic SMPs, the hard segment with the highest phase transition temperature (*T*_perm_) stabilizes the permanent shape, whereas the soft segment with lower transition temperature (*T*_trans_) allows the material to deform and enables fixation of the temporary shape. During a typical shape memory cycle, the permanent shape is first deformed into a temporary shape under mechanical loading at a temperature above *T*_trans_ of the SMP. This temporary shape can be fixed indefinitely by cooling the sample to the temperature below *T*_trans_, followed by releasing the mechanical loading. Increasing the temperature above *T*_trans_ enables the SMP to soften and to restore its original shape.

There has been much effort to investigate the bulk formulation of SMPs, macroscopic deformation, and the bulk material recovery mechanism^2^,^11^,^12^,^13^. It is only recently that researchers begin to study the deformation and recoverability of surface-patterned SMP and to generate stimuli-responsive surfaces with switchable properties^9^,^14^,^15^,^16^. Surface pattern can be described by geometric parameters, including feature size^17^,^18^, spacing^15^, shape^19^, and lattice symmetry^20^,^21^. Few reports have shown the deformation of micropatterns in SMPs, such as micropillars, microprotrusions and microwrinkles, by utilizing the large modulus change near the phase transition temperature^9^,^22^,^23^. Reddy *et al.*^9^ reported that the on-off adhesion process of a SMP micropillar structure was realized with tilted arrangement of the pillars mediated by a force load and dragging steps. Chen *et al.*^15^ exploited the shear deformation and recovery of high-aspect-ratio SMP micropillars to manipulate the surface wettability, whereby distinct wettabilities in the tilted state and the recovered vertical state were demonstrated, especially at larger pillar spacing. In their shape recovery study, it was found that all the pillars in a square array recovered uniformly; while for the relatively close-packed hexagonal array, the recovery was more localized due to lateral contact between the tilted pillars. Xu *et al.*^16^ showed that deformation via hot pressing or stretching of micro-optics (such as microlens, microprisms) resulted in the transformation of the microscopic surface features, and thus changing the optical transmission. The above examples show an increasing interest in exploring adaptive surface patterns for unique applications. However, there is a lack of study on the effects of surface geometries on the deformation and recovery of SMP especially at the micro- and nanometre length scales.

The physical integrity of the surface textures has a direct consequence on the stability of the properties associated with the textures. While studies of mechanically deformable surface patterns on polymers have been reported^24^,^25^,^26^, there is a dearth of comprehensive experimentally-verified study on correlating geometric designs of surface textures with pattern deformation and recoverability. It remains to be seen how length scale and pattern design of a structured surface influence the pattern deformation/recovery and contribute to the associated responsive functions, and hence the rational design of responsive surfaces remains a challenge. Furthermore, as geometries of a matter change from bulk to micro- and nanometre length scale, the effects of increasing surface area per volume can be pronounced. It is of interest to find out whether the deformed pattern can be completely recovered as the contribution of surface energy becomes increasingly dominant at nanoscale. The aim of this work was therefore to systematically investigate the effects of geometry and size on the deformation (mediated by tensile strains) and recovery of surface textures in a shape memory thermoplastic elastomer polyetherurethane. Herein, the degree of deformation and recovery were quantitatively investigated for surface structures with a range of length scales (2 μm- vs. 200 nm-gratings) and feature designs (anisotropic grating vs. isotropic pillars, and protruding pillars vs. recessed holes) fabricated via thermal nanoimprint lithography (NIL). NIL is a simple and reliable fabrication approach capable of creating sub-10 nm features at low cost^27^. The deformed and recovered micro- and nanopatterns were subsequently used for the study of surface wettability. Rational geometric design of shape memory surface pattern could be applied to create a reconfigurable surface where the wettability could be tuned reversibly.

## Results and Discussion

### Imprinted structures and thermomechanical cycle of shape memory polymer

**Figure 1:**
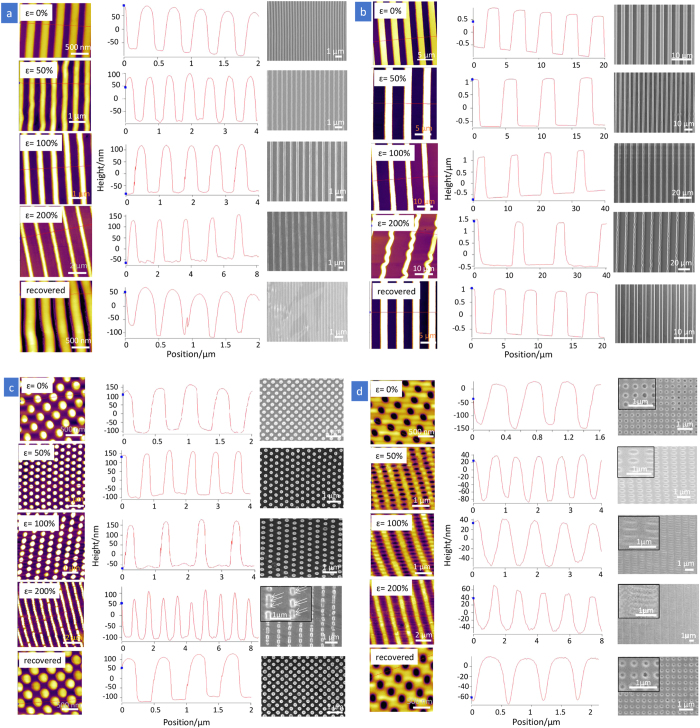
Morphological analyses of original pattern, deformed pattern under different tensile strains and recovered pattern. (**a**) 200 nm-grating, (**b**) 2 μm-grating, (**c**) 250 nm-pillars and (**d**) 200 nm-holes. Left: topographic AFM images; Centre: corresponding height profiles from AFM images; Right: top-view SEM images. Note that the scales for x- and y-axes in the height profiles of AFM are different so as to clearly present the values of dimensions on surface topographies.

**Figure 2:**
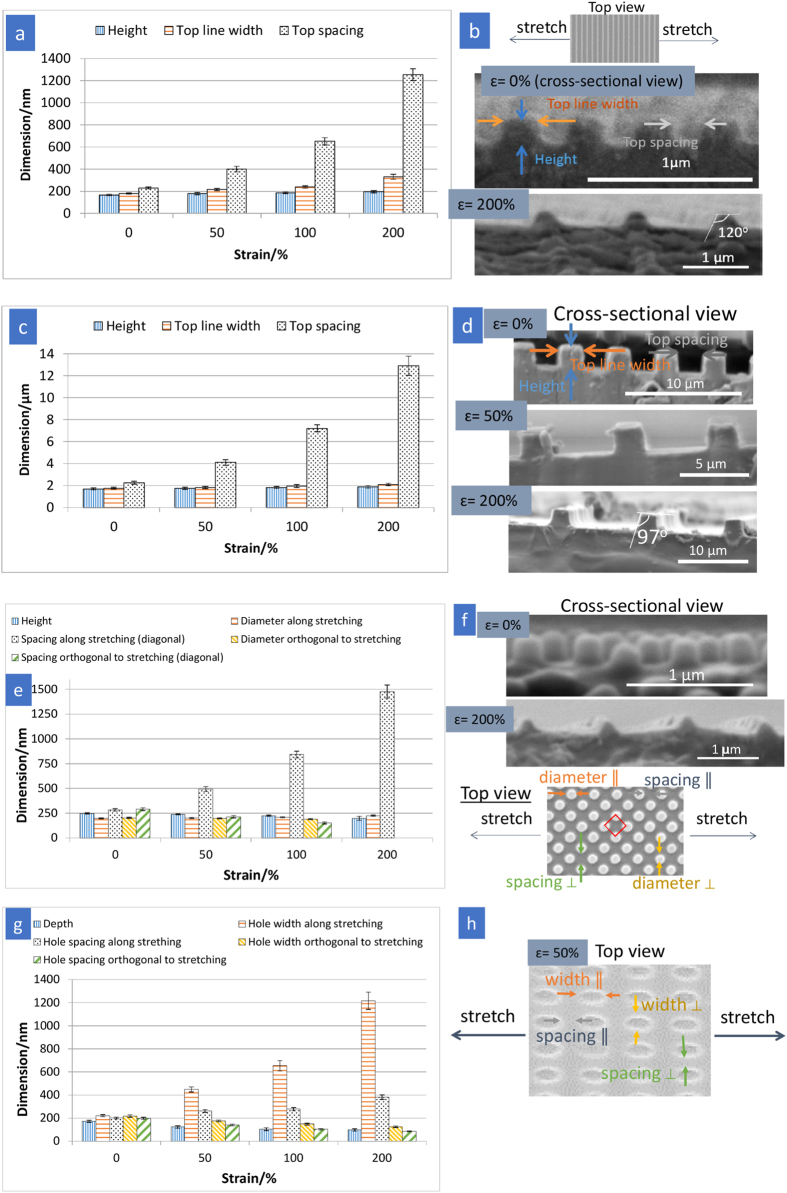
Dimensional analyses (mean ± standard deviation for *n* > 60 measurements) of surface patterns under different tensile strains. (**a**) 200 nm-grating, (**c**) 2 μm-grating, (**e**) 250 nm-pillars and (**g**) 200 nm-holes, and (**b**,**d**,**f**,**h**) associated specifications of surface-patterned samples, including the top and cross-sectional views of surface textures and illustration of stretching direction. For grating structure (**a**–**d**), the line width was measured at the tops of the gratings and is denoted as “top line width”. The measurement of the top part of gratings also applies to the spacing between lines, which is denoted as “top spacing”.

The thermoplastic SMP Tecoflex EG72D used in this work is a cycloaliphatic polyetherurethane block copolymer comprising two phase-segregated components: hard segments composed of methylene bis(*p*-cyclohexyl isocyanate) and 1,4-butanediol, and soft segments composed of poly(tetramethylene glycol) (Supplementary Fig. S1)^12^. In a dynamic mechanical analysis, the glass transition temperature (T_g_) of the soft and hard segments were determined to be 74 °C (*T*_trans_) and 120–140 °C (*T*_perm_), respectively^12^,^28^. Imprinting the Tecoflex film at 190 °C (above *T*_perm_) resulted in a permanent shape change. The AFM and SEM images of the as-imprinted structures, including a 200 nm-grating array, a 2 μm-grating array, a 250 nm-pillar array and a 200 nm-hole array, are shown in Fig. 1a–d (applied strain, *ɛ* = 0%). Based on AFM and SEM images, the dimensions of the surface structures were measured and compared with the dimension of the Si mould surface relief structures. For all the samples, the yield of pattern transfer into Tecoflex films was nearly 100%. The nanoimprinted Tecoflex surface structures (*ɛ* = 0%) replicated closely the dimension of the Si mould. While the tops of 2 μm-grating were flat (Figs 1b and 2d), the tops of the 200 nm-grating and 250 nm-pillars were in the form of round shape (Figs 1a,c and 2b,f), which could be associated with incomplete filling of the mould during imprinting. Incomplete filling is a phenomenon commonly encountered in polymer flow in small cavities, arising from the competition between capillary flow and pressure flow^29^. While incomplete filling may be overcome through further process optimization, these initial profiles of the micro- and nanostructures do not affect the deformation and recovery studies, hence the profiles of the 200 nm-grating and 250 nm-pillars were accepted as the permanent structures. Note that the measurements of these surface textures were done using cross-sectional views of AFM and SEM images, instead of top-view measurement.

Tension (stretching) was applied as a mechanical stimulus to deform the imprinted polymer films at three different macroscopic tensile strains (i.e. 50(±5)%, 100(±7)% and 200(±15)%) (mean ± s.d.). The macroscopic tensile strain refers to the change in overall length over original length of the film (20 mm). During deformation, the permanent shape of the patterned Tecoflex film was first elongated into a temporary state under mechanical tension at 90 °C, which is higher than *T*_trans_ but lower than *T*_perm_. At temperature above *T*_trans_, the soft segments are in a highly mobile and flexible rubbery state with a reported Young’s modulus of 7 MPa at 80 °C^12^. The mechanical stretching deforms and aligns the polymer chains into a higher energy state with lower entropic freedom. The elongated sample was then cooled to the temperature below *T*_trans_, and the flexibility of the soft segments is greatly reduced with a reported higher Young’s modulus of 400 MPa at 25 °C^12^, resulting in a fixation of the temporary pattern. The sample was then unloaded at room temperature, and a slight contraction as a result of the elastic recovery was typically observed. Reheating the material above *T*_trans_ without mechanical constraints recovered the permanent surface pattern as a result of system energy minimization and shape memory effect.

### Effects of geometric designs on surface pattern deformation and recovery

Figure 1a–d shows the AFM and SEM images of the surface patterns under different tensile strains and after recovery. The corresponding dimensional changes of surface patterns and the illustrations showing the stretching directions for various surface patterns are presented in Fig. 2. The dimensional changes of the surface pattern were obtained from AFM line profiles and SEM images near the centre of the neck region in the stretched samples. The necking effect of the bulk film further causes the strains in the stretching direction exhibited by the line spacing (for grating), pillar spacing and the width of the major axes of the ellipse (for holes) to be much larger than the overall macroscopic tensile strain. For example, on 200 nm-grating, as the overall stretching increases from 50% to 200%, the strain on the line spacing increases from 74% to 445%. In this study, we analysed the measureable dimensions in terms of height, width and feature spacing separately to compare the pattern deformation and shape recovery in pairs of patterns: 1) micrometre and nanometre gratings 2) anisotropic and isotropic patterns 3) protruding and recessed structures.

#### Length scale of surface texture (200 nm- vs. 2 μm-gratings)

**Figure 3:**
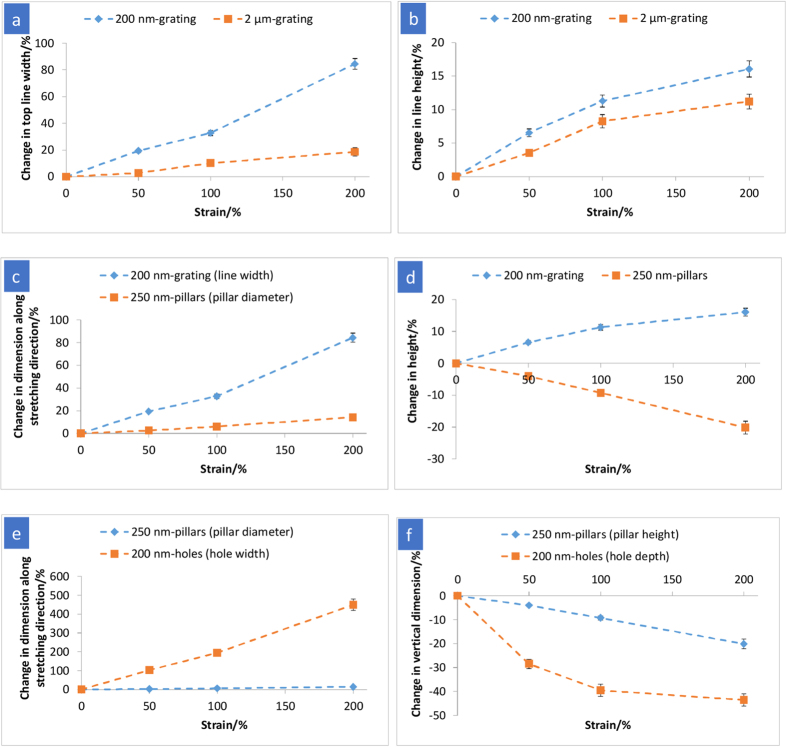
Quantitative dimensional changes (%) in surface pattern as a function of tensile strain. The dashed lines are to guide the vision. (**a**,**b**) 200 nm-grating vs. 2 μm-grating, (**c**,**d**) 200 nm-grating vs. 250 nm-pillars and (**e**,**f**) 250 nm-pillars vs. 200 nm-holes. Positive and negative signs on the y-axis represent a dimensional increase and decrease, respectively.

Variation in length scale of gratings (200 nm vs. 2 μm line width, both at aspect ratio of 1:1 and duty cycle of 1:2) was compared. The line width and spacing increased with increasing levels of stretching, as expected, when the films were stretched in the direction orthogonal to the longitudinal axis of the grating (Fig. 2a–d). While a uniform deformation in the transverse direction is normally expected for a planar bulk film under uniaxial tension, the 2 μm- and 200 nm-gratings were observed to undergo non-uniform deformation as a result of stretching. On the protrusion, there is a decreasing effective tensile stress (strain) from the base (bulk of the film) to the top part of the grating, as demonstrated by the tapered cross-sectional profile in the SEM images (Fig. 2b,d) of the 200 nm- and 2 μm-gratings (*ɛ* = 200%). Therefore, the line width presented in Fig. 2a,c refers to the width of the tops of the gratings and is denoted as “top line width”. The measurement of the top part of gratings also applies to the spacing between lines, which is denoted as “top spacing”. The deformation of the line width was observed to be lower than the macroscopic tensile strain for both 200 nm- and 2 μm-gratings, as shown in Fig. 3a. This observation concurs with Xu *et al.*^16^, who reported that for microprisms the maximum principle logarithmic strain modelled by finite element analysis reached 200% for an overall stretching of 400% and it decreased from the bottom to its top part. Similar results were obtained because both protruding patterns were deformed in response to stretching, where the applied stress was directly distributed over the entire plane of the bulk film, rather than the protruding parts. Figure 3a shows the larger deformation in the 200 nm-grating, as compared with 2 μm-grating. This may be caused by a number of factors such as the higher free surface-to-volume for the 200 nm-grating that results in a higher proportion of polymer chains with increased mobility on the surfaces. Enhanced polymer chain mobility near free surfaces has been evidenced from a reduction in glass transition temperature of ultra-thin polymer films, where the high surface area-to-volume ratio results in a larger free surface^30^,^31^. Furthermore, the surface tension induced stress would play a role in the enhancement of chain mobility on the surface^32^,^33^. As the line width decreases from 2 μm to 200 nm-gratings, the stress caused by surface tension increases. Qualitatively, when the stress exceeded a critical value, non-Newtonian behaviour at highly flexible and mobile state (above *T*_trans_) may be associated with a reduction in apparent viscosity. In the present work, the line width of 200 nm-grating increased drastically for macroscopic applied strain of 200% (Fig. 3a). This finding could be attributed to the dependence of surface pattern deformation on its underlying bulk film’s properties. At *T*_trans_, bulk Tecoflex® EG72D had been reported to undergo nonlinear hyperelastic response under uniaxial tension, in which a drastic decrease in the slope of stress-strain curve was observed for *ɛ* > 100%^28^. As the material begins to harden by a smaller proportion at *ɛ* > 100%, the large decrease in cross-sectional area of the bulk film at higher applied strains would cause more stresses to transfer and propagate continuously along the nanograting, enhancing the deformation of 200 nm-grating width. This is in contrast to 2 μm-grating with a lower proportion of mobile polymer chains on the surface, which restricts the deformation of surface pattern in response to bulk deformation.

As the gratings were stretched, it was anticipated that the dimension along the stretching direction (grating line width) would increase and the line height would decrease to compensate for the increased strain in the stretching direction due to a positive Poisson’s ratio^34^. Instead, as shown in Fig. 3b, the line height linearly increased as the tensile strain increased from 0% to 100%, and appeared to be reaching a plateau when the applied strain increased to 200%. The increase in line height as a function of macroscopic strain is against the bulk properties of the polymer. While a robust mathematical or modelling description of the geometry dependence of pattern deformation is beyond the scope of this work, the basic phenomenological process may be understood as follows. AFM was used to analyse the surface topography of gratings under mechanical loading *in situ*. The height of the 200 nm-grating (with original height of 168 ± 6 nm, mean ± s.d.) that had been stretched to a macroscopic strain of 130% above *T*_trans_ was measured to be 165 ± 5 nm (which is comparable with the original height (P = 0.54)) after cooling to room temperature (while still under loading) (Supplementary Fig. S2). The insignificant change in line height upon stretching indicates a non-uniform stress distribution, where stress along the stretching direction is much greater than the stress transverse to the stretching. This material response can be explained by considering the directional flexibility of polymer chains and applied strain rate under uniaxial tension. The polymer chains are relatively more constrained in the direction perpendicular to the stretching path, while they move and realign along the stretching direction. In addition, since the strain rate in the stretching experiment was considerably high (~200% min^−1^), the polymer might exhibit an increased resistance to transverse deformation due to the insufficient time for molecular movement and relaxation. Subsequently, upon unloading, some strain along the stretching direction was rebounded to an equilibrium strain of ~100% and the line height was found to increase to 187 ± 8 nm (Fig. 2a), likely a result of outward stress induced in the transverse direction to the contraction. On the other hand, when stretched at a considerably high macroscopic strain (≥200%) above *T*_trans_, as explained by a similar line of reasoning as the large deformation of line width described earlier, more stresses were experienced by the grating, decreasing the line height to 155 ± 4 nm (Supplementary Fig. S3). Subsequently, upon unloading process at low temperature, the increased line height due to a slight contraction of the elongated sample (from 250% to an equilibrium strain of ~200%) compensated for the initial decrease, thus plateauing the overall increase in line height.

**Figure 4:**
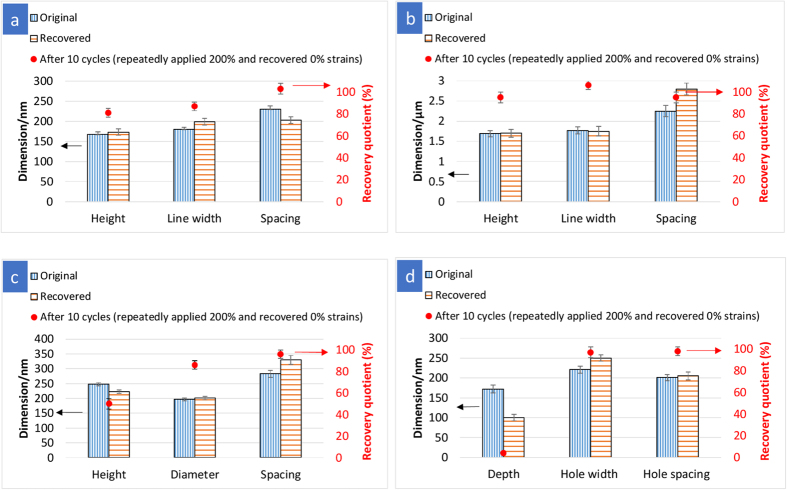
Geometric dimensions (mean ± standard deviation for *n* > 60 measurements) of the original and recovered patterns. (**a**) 200 nm-grating, (**b**) 2 μm-grating, (**c**) 250 nm-pillars and (**d**) 200 nm-holes, and the corresponding calculated recovery quotients after 10 cycles of repeated deformation at 200% strain and thermal recovery.

By reheating the material above *T*_trans_ without any mechanical constraint, the SMP recovers their permanent shapes. The macroscopic recovery determined by measuring the length of the film is 90–100% in all cases. The recovery is driven by the soft segments gaining entropy by moving from an aligned state to a random coil^35^,^36^. In the present study, no strain-holding was applied above *T*_trans_ during deformation process so as to prevent the surface features from being erased at elevated temperatures^16^,^33^. In the absence of strain-holding, reorientation and realignment of the polymer chains during deformation is less extensive, and hence the original bulk permanent shape can recover almost completely^37^. In this study, AFM and SEM measurements were used to assess the degree of recovery for the surface patterns. The recovery of the original shape was quantified by defining a recovery quotient, *R* = (*L*_temporary_ − *L*_recovered_)/(*L*_temporary_ − *L*_permanent_), where *L* represents the length in the original (*L*_permanent_), deformed (*L*_temporary_) and recovered (*L*_recovered_) states^12^. The measurement was carried out by taking the average values of the dimensions over at least 10 different locations on the same pattern. The original and recovered dimensions of a pattern as well as the corresponding recovery quotient after 10 cycles of repeated deformation at 200% strain and thermal recovery are shown in Fig. 4. For the 250 nm-grating, the line height and width showed a recovery quotients of ~85% while the spacing recovered nearly 100% (Fig. 4a). This variation in recovery quotients was possibly due to the difficulty in measuring the dimensions of small features on the same spot, rather than the intrinsic inferior recovery. On the other hand, the 2 μm-grating showed nearly 100% recover in all dimensions (Fig. 4b). The recovery of grating structure could be caused by entropically-driven shape memory effect.

#### Anisotropic 200 nm-grating vs. isotropic 250 nm-pillars

The 250 nm-pillar-structured films were stretched diagonally along the lattice (Fig. 2f). This is to ensure that the duty cycle of 1:2 along the stretching direction was comparable to that of the 200 nm-grating. As shown in Fig. 3c, the 250 nm-pillars were elongated along the stretching direction, although to a lesser extent compared with the strain exhibited by the grating line width. The 200 nm-grating imparts an anisotropic effect to the deformation; stress would propagate primarily along the continuous lines of the nanograting as a result of intra- and interchain interactions within the polymer network, which would in turn lead to a larger strain in the stretching direction. Although the original 250 nm-pillars are in an isotropic array on the surface, stretching was found to result in an anisotropic arrangement as shown by the changes in the pillar-to-pillar spacing. In the direction orthogonal to stretching, the spacing between pillars decreases when the strain varies from 50% to 200% (Figs 1c and 2e). At the strain of 200% (Fig. 1c) where arrows in the inset show that some pillars make contact with each other, the spacing between pillars greatly reduced, and the pillar diameter and spacing orthogonal to the stretching direction became non-measureable.

Interestingly, the heights of the gratings and pillars increased and decreased, respectively, with increasing applied strains (Fig. 3d). For the *in situ* analysis, the height of 250 nm-pillars that had been stretched to a macroscopic strain of 260% above *T*_trans_ was found to have reduced quite substantially to 156 ± 10 nm from 248 ± 5 nm (Supplementary Fig. S4) after cooling to room temperature. Upon unloading at room temperature, only a small elastic recovery in pillar height was observed as a consequence of over stressing during the deformation step. Surface-to-volume ratio was deemed to govern the deformation of these nanopatterned surfaces. The theoretical surface area-to-volume ratios for the 200 nm-grating and 250 nm-pillars were calculated to be 0.017 and 0.024 nm^−1^, respectively, based on the actual pattern dimensions measured from their AFM and SEM images. Higher surface area-to-volume ratio for the 250 nm-pillars results in a greater material’s deformation response when stretched above *T*_trans_, likely due to a higher proportion of mobile polymer chains on the surface. Enhanced relaxation (segmental or molecular rearrangement) and chain dynamics could be the additional contributions to promote material response as pillars present unconstrained geometries^33^.

It had been reported that the macroscopic bulk recovery is mainly dependent on the applied strain during shape programming step, whereby a higher strain would reduce the recovery as a result of extensive reorientation of the molecular network and irreversible plastic strain^28^,^36^. In this paper, we found that although the same strain was applied and the pattern deformation occurred at a relatively low strain, the recovery of nanopatterned surfaces with different geometries varied. For 250 nm-pillars (Fig. 4c), although the structure restored its original pillar diameter and spacing (*R* ~ 90%) during reheating, the pillar height did not completely recover (*R* = 50%). The pillar height was found to reduce during pattern deformation. In order to recover its original shape, the pillar height has to increase, leading to a high energetic requirement on the material. It had been reported that thermoplastic polymers have the tendency to smooth out their surfaces (i.e. surface levelling) to minimize the surface energy at elevated temperatures^14^,^38^,^39^. Therefore, in contrast to bulk recovery, the surface pattern recovery mechanism could be driven by the interplay of the surface area minimization and entropy elasticity of the soft segment. The 50% recovery of the 250 nm-pillar height could result from the competing effects of the strain recovery that favours the original state and the surface energy minimization that prefers the surface levelling. Unlike the 250 nm-pillars, the height of 200 nm-grating was found to increase as a result of stretching, as discussed earlier (Fig. 3b). During recovery, their height at deformed state (195 ± 10 nm) had to reduce (173 ± 8 nm at recovered state), which is close to the original height of 168 ± 6 nm without further increasing the surface area. Therefore, a relatively high recovery quotient was observed for the height of 200 nm-grating (Fig. 4a).

#### Protruding 250 nm-pillar vs. recessed 200 nm-hole arrays

The pattern responses and shape recovery of the recessed (hole) structure were compared with the protruding (pillar) structure. The hole structure was elongated along the stretching direction (in parallel with lattice vector) (Fig. 2h), and the circular holes were deformed to an array of elliptical slits (Fig. 1d). The change in hole width (width of major axes) with the levels of stretching was more pronounced than that in pillar diameter (Fig. 3e). Since the holes are embedded within the polymer film for the recessed structure, the applied stress is distributed over the entire plane of the film and the deformation of bulk film is manifested in hole shape deformation. For pillars, there is a non-uniform stress field with decreasing effective tensile strain (stress) from the bottom to the top of the pillar (Fig. 2f, *ɛ* = 200%). For holes, applying macroscopic strains results in hoop stress (circumferential), perpendicular axial stress and radial stress. In the region of radial stress (the continuous solid surrounding the holes), stresses in the opposite direction exerted by the adjacent holes would affect the deformation of hole array. Therefore, the spacing variation between holes may be an important parameter that affects the hole deformation; to fully understand this phenomenon would be a subject of a future investigation. On the other hand, when the 200 nm-hole array is stretched, it contracts in the directions transverse to the stretching due to a positive Poisson’s ratio^34^, hence reducing the hole depth. Recessed (hole) structure exhibited a more significant change in the hole depth, in comparison with that in the pillar height (Fig. 3f). This is again because the stress is absorbed in-plane by the entire film where the holes are essentially the free surfaces embedded in the film. The holes showed a nonlinear depth reduction with applied tensile strains, which could probably be associated with a nonlinear dependence of the Poisson’s ratio on longitudinal strain proposed by the strain energy function of Ogden on hyperelastic materials^40^.

For the recovery of 200 nm-hole array (Fig. 4d), the hole diameter was restored almost completely. However, the hole depth failed to recover (*R* = 4%), which is even lower than the recovery quotient of pillar height (*R* = 50%). As observed from the hole deformation during shape programming, the hole depth was significantly decreased (Fig. 3f), which favours the minimization of surface area. Therefore, the contribution of surface energy increases relative to elastic recovery energy, resulting in an incomplete recovery of hole depth through maintaining the shallower hole depth^41^. This finding manifests the importance of studying the pattern deformation as a function of surface geometry under the same applied strain, as it would influence the subsequent shape recovery.

### Pattern transformation-induced wettability changes

**Figure 5:**
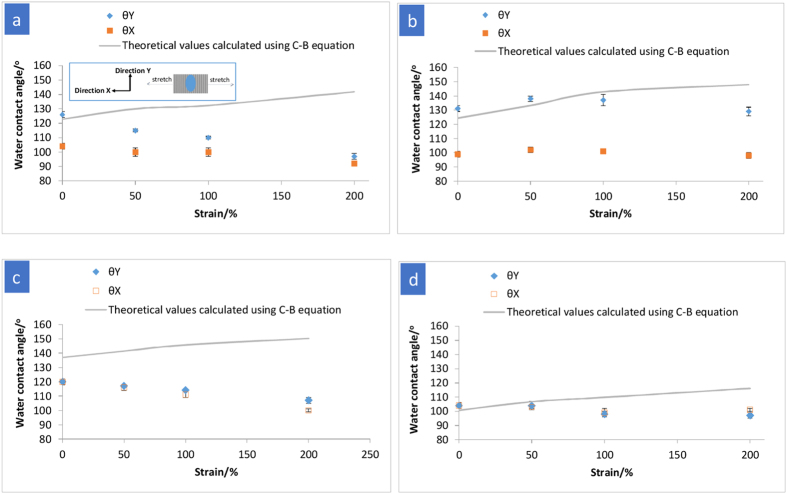
The dependence of water contact angles (CAs) (*n* > 5 measurements on the centre of the neck region) on the surface patterns as a function of tensile strain. (**a**) 200 nm-grating, (**b**) 2 μm-grating, (**c**) 250 nm-pillars and (**d**) 200 nm-holes, and theoretical prediction by the Cassie-Baxter model. *X*-direction and *Y*-direction are defined as the directions along stretching and orthogonal to stretching, respectively. *θ*_X_ and *θ*_Y_ are the static CAs measured in the *X*-direction and *Y*-direction, respectively.

Changes in surface wettability are associated with changes in surface pattern, hence a deformable surface pattern opens up a possibility of a transformable surface wetting control. Surface wetting as a function of topography often follows the classical Cassie-Baxter (C-B) or Wenzel models^42^,^43^. The equilibrium contact angle on a flat Tecoflex film was measured to be 89° ± 1° (mean ± s.d.), and surface texturing may result in the surface to become either more hydrophobic or more hydrophilic. Surface wetting measured by water contact angle (CA) is highly dependent on the directionality on surface texture. Anisotropic surface textures result in anisotropic wetting characteristic as shown in the 200 nm- and 2 μm-gratings (Fig. 5a,b). In an anisotropic wetting, a substantially different water CA is observed at viewing directions 90° from each other. In this study, *X*-direction and *Y*-direction are defined as the directions orthogonal to (or along stretching direction) and parallel with (or orthogonal to stretching direction) the longitudinal axis of the grating, respectively, with *θ*_X_ as the static CA measured in the *X*-direction, and *θ*_Y_ as the static CA measured in the *Y*-direction.

In the Cassie-Baxter model, the droplet sits on the peaks of the roughened surface and with trapped air below^42^,^44^. The apparent Cassie-Baxter contact angle, *θ*_CB_, is described as follows,


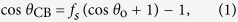


where *f*_s_ is the fraction of the liquid droplet surface in contact with the solid and *θ*_o_ is the intrinsic CA of the material on its flat surface. The solid fraction *f*_s_ was calculated based on the actual pattern dimensions measured from the AFM and SEM images (see Supplementary Information for *f*_s_ calculation). As shown in Fig. 5, the CAs (*θ*_Y_) of as-imprinted, non-deformed surface patterns generally agreed with that predicted by C-B equation except for the 250 nm pillars. The CA on the as-imprinted 250 nm-pillars was observed to be appreciably lower than the CA value calculated using the C-B equation, in this case rounded-top pillars would promote water droplet to penetrate into the void space and sagging occurs at the liquid–air interface^45^.

**Figure 6:**
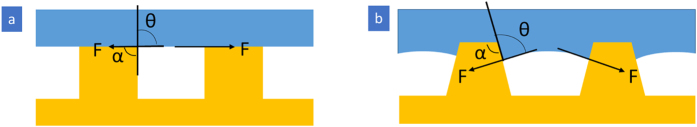
Schematic diagrams (not to scale) illustrating possible solid-liquid-vapour interfaces with the same equilibrium contact angle (*θ*), but different geometric angles (*α*). (**a**) *θ* ≈ *α* and (**b**) *θ* < *α*.

The deformed micro- and nanopatterns were subsequently investigated for the surface wetting properties. On the non-patterned film (as a control group), the CA was found relatively unchanged (91° ± 2°) for the stretched Tecoflex film (*ɛ* = 200%) relative to the unstretched film (89° ± 1°) (Supplementary Fig. S5). While hard segments in the polymer chains would migrate to the surface during stretching^46^, the molecular reorientation did not result in a significant change in the surface wettability in our case. Therefore, the changes in CAs on the surface patterns under different tensile strains observed in our study could mainly be attributed to the pattern transformation, rather than surface chemistry. The CA measurement data for various surface patterns as a function of tensile strain is shown in Fig. 5. Depending on the geometric feature, the CA on a patterned surface varied in different extents with the mechanical strain levels. As shown in Fig. 5a, for the 200 nm-grating, the *θ*_Y_ decreased from 126° to 97° as the tensile strain increased from 0% to 200%. To illustrate qualitatively, we consider a schematic shown in Fig. 6, which depicts solid-liquid-vapour interfaces for a water droplet with the same equilibrium contact angle *θ* of 89° ± 1° on two different patterned surfaces. A local geometric angle, *α*, defines the shape of the solid pattern in contact with water. The as-imprinted 200 nm-grating has an *α* value of ~90° (Figs 2b and 6a), while the deformed 200 nm-grating (*ɛ* = 200%) has a trapezoidal shape with an *α* value of 120° (Figs 2b and 6b). When *θ* < *α* (Fig. 6b), the net force on the liquid-vapour interface is directed downward^47^, promoting the wetting of the texture sidewalls and reducing CA. Since the SMP used is intrinsically hydrophobic, the water droplet would not be able to fully intrude into the patterned structure^48^. Rather, there would be small air spaces trapped within the grooves. Furthermore, the degree of anisotropic wetting (*θ*_Y_ − *θ*_X_) was observed to reduce with increasing the strain level (e.g. 22° at 0% strain vs. 5° at 200% strain). When the length scale is one order of magnitude larger (from 200 nm-grating to 2 μm-grating), the *θ*_Y_ on 2 μm-grating increased (P = 0.01) when changing *ɛ* from 0% to 50% due to lower solid fractions (obeying C-B state). At this low strain (*ɛ* = 50%), the local geometric angle of the texture (*α*) was observed to be ~90° (*θ* close to *α*), as shown in Fig. 2d, hence the liquid-vapour interface recedes to the top of the grating, forming a composite solid-liquid-air interface (Fig. 6a). The *θ*_Y_ began to decrease marginally at high strain (beyond *ɛ* = 100%) (Fig. 5b). This moderate decrease in CA could be attributed to the relatively low deformation of 2 μm-grating (*α* = 97° for 200% strained sample, Fig. 2d), which prevents the water droplet from wetting extensively the grating sidewall. For the 250 nm-pillars, the increased anisotropic pillar arrangement at *ɛ* = 200% (Fig. 1c) could account for the increased degree of anisotropic wetting (8° for *ɛ* = 200% compared to 0° for *ɛ* = 0%) (Fig. 5c). In contrast to the above discussion, the surface wetting characteristic of the holes did not exhibit a systematic relationship between the deformed geometries and the corresponding CAs. The CA on the 200 nm-hole structure remained relatively unchanged with stretching (Fig. 5d). This could be associated with the presence of continuous solid surrounding the holes, which hinder the water droplet from wetting the sidewall.

**Figure 7:**
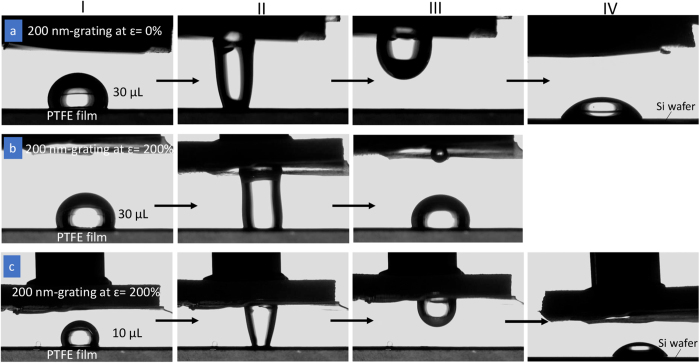
Microdroplet transfer. (**a**) Transfer of a water droplet (30 μL) from superhydrophobic PTFE film to a 200 nm-grating film at original (*ɛ* = 0%) state, and then to a third surface with a much lower CA (Si wafer). (**b**) Failure to transfer a water droplet (30 μL) to the deformed (*ɛ* = 200%) 200 nm-grating. (**c**) When the volume of water droplet is reduced to 10 μL, a successful transfer of the microdroplet between the PTFE film and the deformed (*ɛ* = 200%) 200 nm-grating and then to a Si wafer is shown. Stage I: A microdroplet is placed on a PTFE film. Stage II: 200 nm-grating film is brought into contact with the water droplet and lifted up. Stage III: For (**a**,**c**), the microdroplet adheres to the 200 nm-grating, whereas the grating failed to pick up the water droplet for (**b**). Stage IV: For (**a**,**c**), the water droplet on the 200 nm-grating can be detached to a third surface (Si wafer).

In conjunction with the recovery of surface patterns, the wetting behaviour of the 200 nm-grating shows good reversibility; switching *θ*_Y_ between 126° and 97° and *θ*_X_ between 104° and 92° occurred with good repeatability for 10 transformation cycles (Supplementary Fig. S6). This switchable wetting without changing materials or needing continuous external stress or energy inputs presents an alternative to applications where dynamic control of liquid wetting is needed, for example in fluidic devices, nanoparticle assembly and water collection^15^,^49^,^50^. The reversibly deformable SMP surface pattern (200 nm-grating) may be used as a “mechanical hand” for transferring microdroplets. Figure 7 conceptually demonstrates the transfer of a microdroplet from one surface to another. As shown in Fig. 7a, when the Tecoflex film with original (*ɛ* = 0%) 200 nm-grating (with the pattern facing downward) was brought into contact with a microdroplet (30 μL) on a superhydrophobic poly(tetrafluoroethylene) (PTFE) (Stage I), the microdroplet could be ‘picked up’ by the 200 nm-grating Tecoflex film (Stages II and III), subsequently this microdroplet on the 200 nm-grating could be detached and transferred to a third surface with a much lower CA (Silicon wafer) (Stage IV). However, the deformed (*ɛ* = 200%) 200 nm-grating failed to pick up the water droplet with the same volume (30 μL) of microdroplet (Fig. 7b); a small volume of droplet was transferred but the bulk of it remained on the PTFE film (Stage III). A successful transfer was achieved when the volume of water droplet was reduced to 10 μL (Fig. 7c). By mechanical modulation, the deformable surface texture can be used to selectively transfer microdroplets of different volumes from one surface to another; and by using SMP the deformation of the surface texture may be kept in a transient stage without resulting in a permanent deformation. Water droplet adhesion is known to be dependent on the geometry and length-scale of the surface textures^51^. As such, coupling the current research results with surface texture design provides a novel approach to manipulate liquid transport.

Although there are many existing examples of surface functionalities (such as wetting) achieved by surface texturing, this study has shown the shape memory effect to not only be effective at a certain rationale design of surface pattern (i.e. nanograting), but also to generate reconfigurable surfaces with switchable wettability. The fundamental origins of surface pattern deformation and recovery are not restricted to the specific thermoplastic elastomer (Tecoflex® EG72D) discussed in this study and the concepts can be extended to the general class of shape memory thermoplastic elastomers and chemically cross-linked elastomers that show phase transition behaviours. For instance, varying contents of the hard and soft segments in a thermoplastic elastomer can lead to different thermomechanical properties (e.g. change in *T*_trans_). The SMPs can still be deformed and recovered in the similar manners above the respective *T*_trans_ at a rubbery state. Besides thermal recovery, the understanding gained from this study can also apply to other external stimulation (e.g. magnetic, pH, light) that have shown compatibility with the SMPs by using different materials. Hence, this work can serve as the basis for future studies investigating the role of different geometries of responsive complex structures (e.g. hierarchical, heterogeneity).

## Conclusion

Polyetherurathane-based SMP was thermally imprinted with permanent shape made up of micro- and nanoscale surface textures. The pattern deformation and recovery can be engineered by tailoring surface texture designs including length scale, degree of anisotropicity and shape (protruding vs. recessed structures). While the applied strain and inherent material properties are responsible for the macroscopic bulk deformation, free surface coupled with proportion of mobile chains, surface area-to-volume ratio, dependence of stress distribution on surface geometry are the factors involved in governing the pattern deformation. Distinct deformations of various pattern geometries under the same applied strain could influence the subsequent recovery. In contrast to bulk behaviours, surface pattern recovery mechanism could be driven by the interplay of the surface area minimization and entropy elasticity of the soft segment. We further reported the control of deformable surface patterns on surface wetting tuning without the need for continuous input of external stresses or energies. Changing the water contact angle of the 200 nm-grating was demonstrated to transfer microdroplet through mechanical straining. Therefore, this new approach would open up an avenue of incorporating shape memory surface pattern into microfluidic devices for manipulating fluid transport.

## Methods

### Materials

Tecoflex® (EG72D), a thermoplastic shape memory polymer, was obtained from Lubrizol (USA). The silicon moulds used to fabricate the micro- and nanoscale surface textures are listed in Table 1.

### Fabrication of micro- and nanostructures via thermal nanoimprint

High-resolution and well-defined surface textures can be controllably fabricated through nanoembossing technique, commonly known as nanoimprinting^52^. Silicon moulds were cleaned using Piranha solution (a 3:1 mixture of 96% sulfuric acid with 30% hydrogen peroxide) at 120 °C for 30 min, rinsed with deionized water, dried in a stream of dry nitrogen, and put in a clean oven at 100 °C for 1 h. The moulds were then exposed to air plasma for 10 min in a plasma cleaner (Harrick Plasma PDC-002), operated at 200 mTorr and a power of 30 W. The moulds were further treated with a fluorosilane release agent (0.1 mL) for 4 h by vapour deposition of 1H, 1H, 2H, 2H-perfluorodecyltrichlorosilane self-assembled monolayer. Note that the silane-treated moulds underwent a self-cleaning imprint on polycarbonate films to further remove the physisorbed silane (if any). This is to ensure that there is no influence of silane on the subsequent contact angle measurements. Tecoflex films were first prepared by melting the pellets on a heating stage (200 °C) under a weight of 500 g. After which, the imprinting was performed on the polymer films using a nanoimprinter (SOLVES thermal nanoimprinter) for 30 min at 190 °C and under elevated pressure of 50 bar to allow the softened polymeric material to flow into the trenches of the mould. Following which, the nanoimprint system was cooled down to the temperature below 50 °C. Upon demoulding, these patterns were formed permanently on the surfaces of the polymer films.

### Mechanical stretching of films and shape programming

The polymer films were placed in a home-built sample holder for uniaxial stretching. For grating-structured films, the stretching direction was orthogonal to the longitudinal axis of the grating. The 250 nm-pillar-structured films were stretched diagonally along the lattice so as to ensure that the duty cycle (ratio of the pillar diameter to the period) of 1:2 along the stretching direction was comparable to that of the grating and hole arrays. For 200 nm-hole-structured films, the stretching direction was parallel with the square lattice vector. Each specimen was stretched to different extent of macroscopic strains at 90 °C, and locking the temporary shape upon cooling to room temperature (25 °C), followed by the removal of external stress. Upon thermal annealing at around 90 °C without mechanical constraints, the deformed structure could be recovered. The stretching and recovery experiments on each surface pattern were done in triplicate.

### Morphological analysis

The morphological analysis of the samples was conducted through a field emission scanning electron microscope (SEM) (JEOL FESEM, JSM 6700F) and atomic force microscope (AFM). All samples were deposited with a thin layer of gold (~8 nm) (Fine Coater, JEOL JFC-1200) under plasma current of 20 mA for 45 s prior to SEM imaging. For every sample batch (*n* = 3), since morphologies were found to be consistent, only one representative SEM image is shown. Dimensions of surface pattern on 2 fields in independent SEM images were analysed with ImageJ software. AFM imaging was conducted under a tapping mode using a commercial Asylum Research MFP3D AFM, equipped with a high-aspect-ratio tip silicon cantilever. For stretched samples, the AFM tip was positioned near the centre of the neck region^46^,^53^. Surface pattern dimensions were provided by the software measurement; the values obtained from the AFM software were also cross-checked with the values measured on the SEM images.

### Water contact angle measurement

Wettabilities of samples were characterized by measuring static water contact angles (CAs) using a sessile drop method on a contact angle goniometer (Kino SL200KS). A deionized water droplet (4 μL) was dispensed gently onto the sample surface using a motorized microsyringe, and a photograph of the water droplet was taken immediately with the goniometer camera. CA values were obtained from the integrated software in the goniometer. For every stretched sample batch (*n* = 3), at least 5 measurements were conducted on the centre of the neck region to give an average value of contact angle in each of two directions: orthogonal to and parallel with the stretching direction.

### Statistical analysis

Comparison of data including texture dimensions and water contact angles obtained from different surface patterns and levels of stretching were conducted using an unpaired two-tailed student’s *t* -test. Statistically significant differences were verified when P ≤ 0.05.

## Additional Information

**How to cite this article**: Lee, W. L. and Low, H. Y. Geometry- and Length Scale-Dependent Deformation and Recovery on Micro- and Nanopatterned Shape Memory Polymer Surfaces. *Sci. Rep.* **6**, 23686; doi: 10.1038/srep23686 (2016).

## Supplementary Material

Supplementary Information

## Figures and Tables

**Table 1:** Structures of the moulds used to fabricate the surface textures.

Imprinted surface texture	Mould (with general tolerances of line width/diameter at ±10%; height at ±15%.)
200 nm-grating	Array of 200 nm wide lines (with 400 nm pitch and a height of 200 nm)
2 μm-grating	Array of 2 μm wide lines (with 4 μm pitch and a height of 2 μm)
250 nm-pillars	Square array of holes (250 nm hole diameter, 250 nm depth and 350 nm pitch, i.e. 245 nm spacing diagonally along the lattice)
200 nm-holes	Square array of pillars (200 nm diameter, 200 nm height, and 400 nm pitch)
