# Supplementary material for: Geometry- and Length Scale-Dependent Deformation and Recovery on Micro- and Nanopatterned Shape Memory Polymer Surfaces
Source: Sci Rep. 2016 Mar 30;6:23686. doi: 10.1038/srep23686 (PMC4812242; doi:10.1038/srep23686)
Supplement: Supplementary Information [file srep23686-s1.pdf]

Supplementary Information

Geometry- and Length Scale-Dependent Deformation and Recovery on Micro- and Nanopatterned Shape Memory Polymer Surfaces

Wei Li Lee[1,2], and Hong Yee Low[1,*]

[1]Engineering Product Development, Singapore University of Technology and Design, 8 Somapah Road, Singapore 487372, Singapore
[2]Department of Materials Science and Engineering, Massachusetts Institute of Technology, 77 Massachusetts Avenue, Cambridge, MA 02139, United States
*hongyee_low@sutd.edu.sg

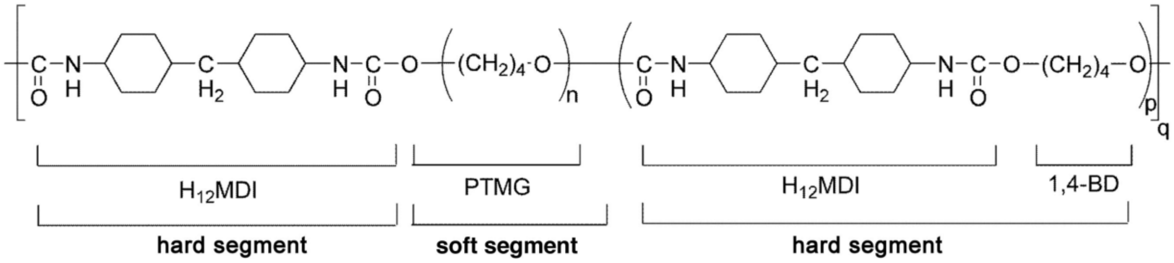

Figure S1. Chemical structure of the shape memory thermoplastic elastomer Tecoflex EG72D. It is synthesized from methylene bis(*p*-cyclohexyl isocyanate) ($H_{12}MDI$), 1,4-butanediol (BD), and poly(tetramethylene glycol) (PTMG).

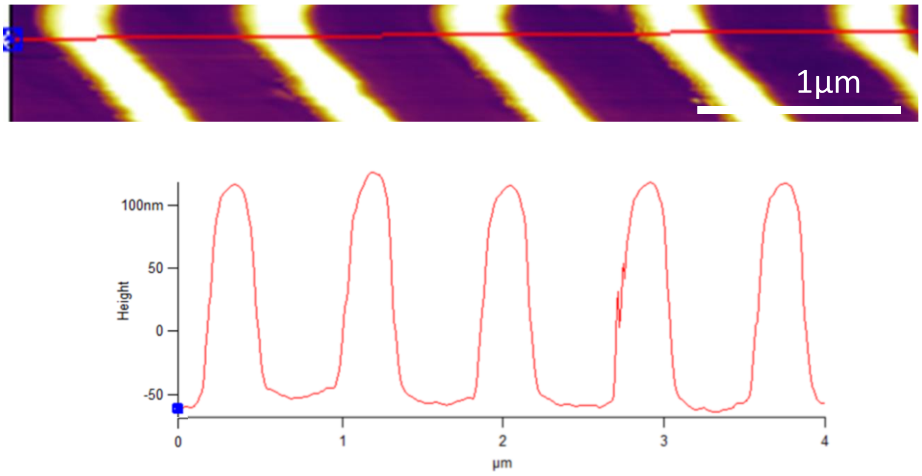

Figure S2. Topographic AFM image and the corresponding height profile of a 200 nm-grating. It had been stretched to an overall strain of 130% after cooling to room temperature (while under loading).

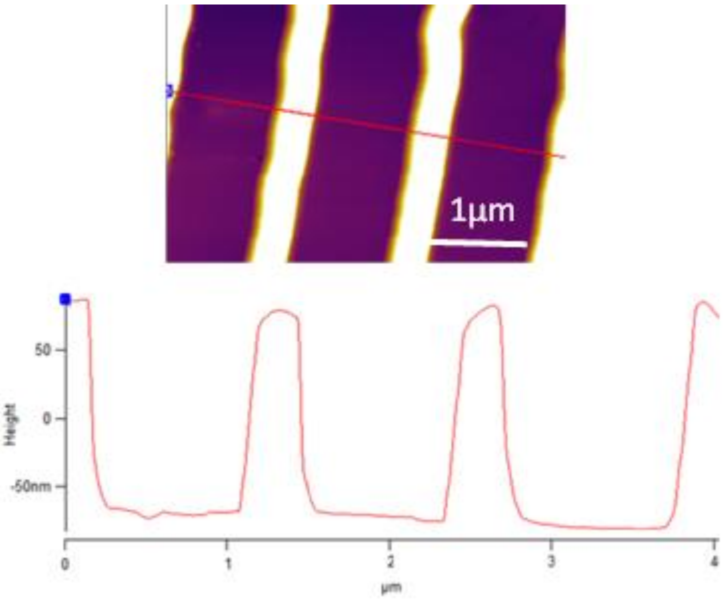

Figure S3. Topographic AFM image and the corresponding height profile of a 200 nm-grating. It had been stretched to an overall strain of 250% after cooling to room temperature (while under loading).

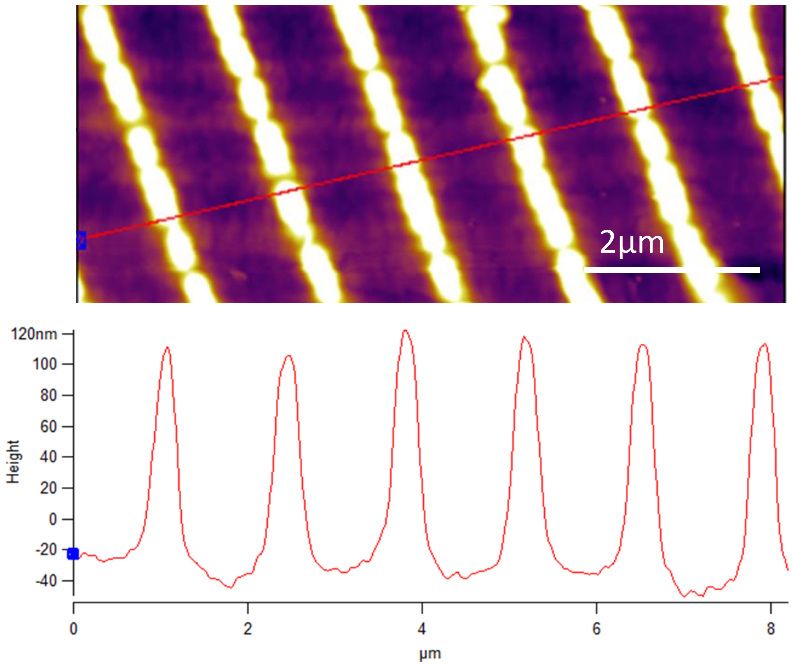

Figure S4. Topographic AFM image and the corresponding height profile of the 250 nm-pillars. It had been stretched to an overall strain of 260% after cooling to room temperature (while under loading).

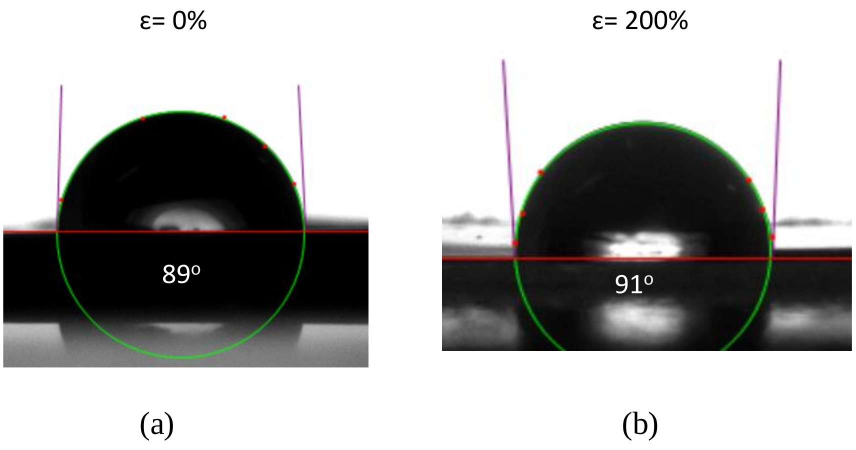

Figure S5. Shape of water droplet on non-patterned Tecoflex films. (a) original ε= 0% and (b) stretched ε= 200%.

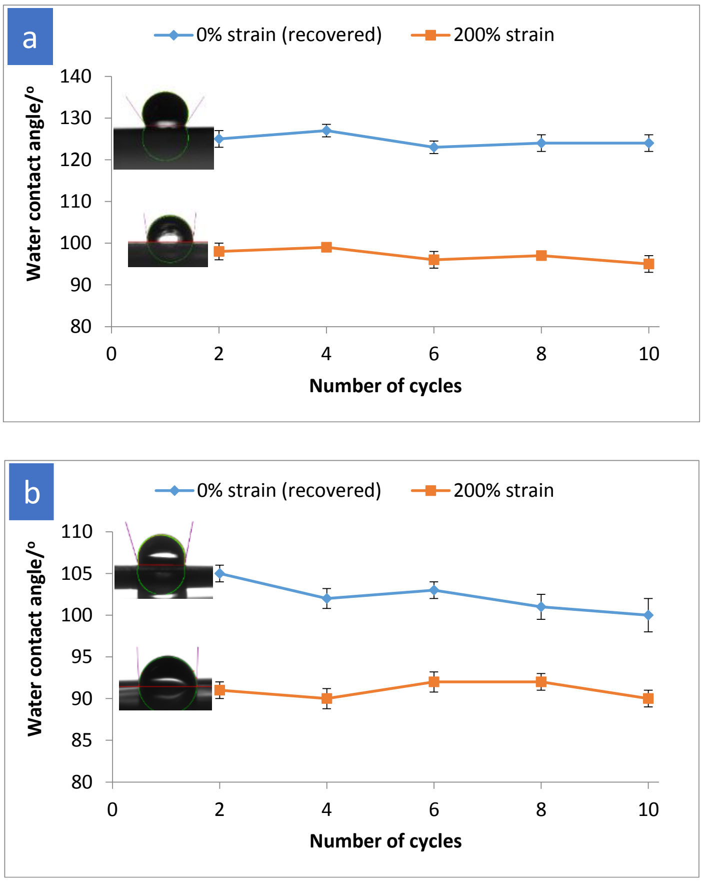

Figure S6. Reversible water wetting function. The CAs of (a) θ_Y and (b) θ_X on the 200 nm-grating as a function of number of cycles of repeated deformation at 200% strain and thermal recovery.

Solid fraction f_s calculation

(a) Grating

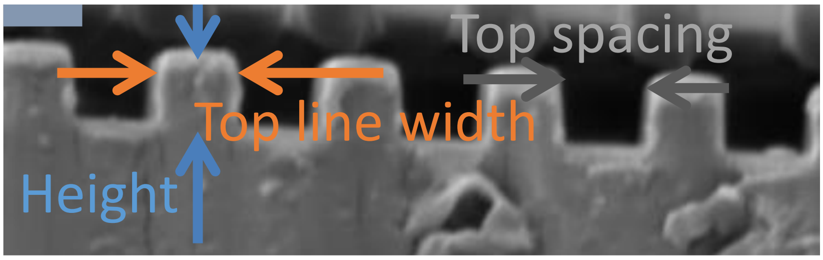

f_s = top line width / (top line width + top spacing)

For example, at ε= 0%, f_s = 180 nm / (180 nm + 230 nm) = 0.44

at ε= 100%, f_s = 239 nm / (239 nm + 652 nm) = 0.27

(b) Pillars

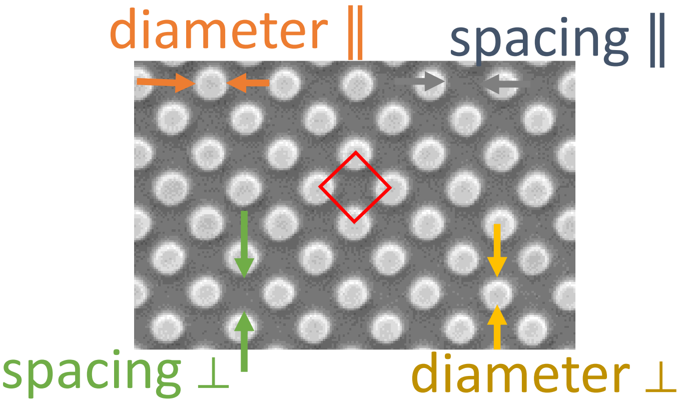

f_s = [π *(diameter∥/2)*(diameter⊥/2)*2] / [(diameter⊥+ spacing⊥)*(diameter∥+ spacing∥)]

For example, at ε= 0%, f_s = [π *(197 nm/2)*(203 nm/2)*2]/[(203 nm + 290 nm)*(197 nm +283 nm)] = 0.27

at ε= 100%, f_s = [π *(209 nm/2)*(191 nm/2)*2]/[(191 nm + 152 nm)*(209 nm +842 nm)] = 0.17

(c) Holes

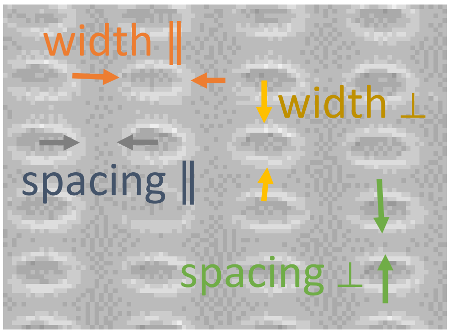

f_s = 1- [π * (width∥/2) * (width ⊥/2)] / [(width ⊥+ spacing ⊥) * (width ∥+spacing ∥)]

For example, at ε= 0%, f_s = 1- [π * (221 nm/2) * (218 nm/2)] / [(218 nm + 198 nm) * (221 nm + 201 nm)] = 0.78

at ε= 100%, f_s = 1- [π * (653 nm/2) * (148 nm/2)] / [(148 nm + 103 nm) * (635 nm + 278 nm)] = 0.67
